# Explicit and implicit information needs of people with depression: a qualitative investigation of problems reported on an online depression support forum

**DOI:** 10.1186/1471-244X-11-88

**Published:** 2011-05-18

**Authors:** Lisa J Barney, Kathleen M Griffiths, Michelle A Banfield

**Affiliations:** 1 Centre for Mental Health Research, Australian National University, Canberra, ACT, 0200, Australia; 2 Australian Primary Health Care Research Institute (APHCRI), Australian National University, Canberra, ACT, 0200, Australia

## Abstract

**Background:**

Health management is impeded when consumers do not possess adequate knowledge about their illness. At a public health level, consumer knowledge about depression is particularly important because depression is highly prevalent and causes substantial disability and burden. However, currently little is known about the information needs of people with depression. This study aimed to investigate the explicit and implicit information needs of users of an online depression support forum.

**Methods:**

A sample of 2680 posts was systematically selected from three discussion forums on an online depression bulletin board (blueboard.anu.edu.au). Data were examined for evidence of requests for information (reflecting explicit needs) and reports of past or current problems (implicit needs). Thematic analysis was conducted using a data-driven inductive approach with the assistance of NVivo 7, and instances of questions and people reporting particular types of problems were recorded.

**Results:**

A total of 134 participants with personal experience of depression contributed to the data analysed. Six broad themes represented participant queries and reported problems: Understanding depression; disclosure and stigma; medication; treatment and services; coping with depression; and comorbid health problems. A variety of specific needs were evident within these broad thematic areas. Some people (n = 46) expressed their information needs by asking direct questions (47 queries) but the majority of needs were expressed implicitly (351 problems) by the 134 participants. The most evident need for information related to coping with depression and its consequences, followed by topics associated with medication, treatment and services.

**Conclusions:**

People with depression have substantial unmet information needs and require strategies to deal with the difficulties they face. They require access to high quality and relevant online resources and professionals; thus, there is a need to rectify current gaps in the provision of information and limitations of dissemination. Greater knowledge about depression and its treatment is also needed at the general community level.

## Background

Health literacy is an important element in the prevention and management of chronic illnesses [1]. Health management is hindered when people with depression do not possess adequate knowledge about their illness. A number of recent studies have explored the information needs of people with illnesses such as cancer, cardiovascular disease, AIDS, diabetes, epilepsy and bipolar disorder (see [2-7]). However, despite the substantial disability and burden of depression [8], there has been little research into the information needs of those with depression.

One study investigated the questions people with depression asked their psychiatrists and found that the most common questions related to the expected duration of medication treatment, time until recovery, and whether they were 'normal' [9]. Little is known though about the broader information needs of people in the community with depression, many of whom do not receive treatment from mental health professionals.

In general, medical professionals are important sources of information for those with health problems. However, many people with depression are reluctant to seek professional help [10] and even those in contact with healthcare services may not receive adequate information [11,12]. Other sources of help and information include complementary and alternative medicine providers, religious advisers, family and friends, support groups and internet resources [13-15]. Online message boards and support groups provide the opportunity to seek information, advice and opinions from other individuals experiencing the same problem and are believed to be an important source of health information for people with a variety of conditions [16,17] including mental health problems [18]. Online resources enable users to access information and interact with others anonymously, a factor which may particularly important for people with stigmatised illnesses. Previous research has found that stigma is a prominent barrier to seeking information for mental health problems and that the Internet is helpful in this regard [19].

Research into the nature of online communication by Miller and Gergen [20] found informative and help-seeking interchanges including *requests for information *on the problem situation, *requests for help *with a personal problem, and *problem disclosure*. The latter involves revealing a personal problem or complaining of shortcomings, stresses and worries in such a way that help is invited, and was found to be one of the most frequently-used forms of dialogue. Although no explicit requests are involved, disclosure of problems appears to be an important means of obtaining help and information.

Accordingly, it appears that information-seeking may be conducted in explicit or implicit ways. Explicit information-seeking is seen in the form of a direct request for information (a question) whereas implicit strategies may be incorporated in more subtle forms of exchange. Considering the major role of problem disclosure as a means of seeking information, investigation of information needs should consider both explicit requests for information and implicit needs as reflected by the problems disclosed.

In order to ascertain the information needs of people with depression in the community, hereafter referred to as '*consumers*', the current study involved a comprehensive examination of the reported problems of and the questions asked by users of an online depression support forum.

## Methods

### Data collection

In 2004 a free-to-the public, online peer-to-peer bulletin board *BlueBoard *(blueboard.anu.edu.au) was developed and attached to the depression information website *BluePages *(bluepages.anu.edu.au). *BlueBoard *was designed for people suffering or in recovery from depression, and for their friends and carers, with the aim of enabling anonymous interaction and mutual support. The board was established and hosted by the Depression and Anxiety Consumer Research Unit at The Australian National University. It was moderated by academic consumer researchers, one of whom was a registered psychologist and took overall responsibility for the board. The moderators did not provide advice or therapy. Potential users became aware of *BlueBoard *through web links and media exposure or search engines. The Board comprised a number of forums for discussion, the majority of which could be browsed regardless of Board membership. However, only registered members could create a topic ('*thread') *of discussion under which they could type comments ('*posts'*), either initiating conversations or responding to others' comments. The Board was asynchronous, the posts appearing sequentially and thus not requiring the simultaneous presence of all participants in the thread.

Participants were recruited into the current project when they joined *BlueBoard*. They were explicitly informed that if they consented to participate, register and post messages to the board their posts would be monitored and analysed for the purposes of research. Participants were required to familiarise themselves with the rules of the Board. For the purpose of minimising distress to others and avoiding the possibility of contagion, participants were not permitted to post material referring explicitly to suicidal thoughts or self-harm actions. Material considered inappropriate was removed by the Board moderators. Participants were asked to use only one login and additional accounts were disabled in any instances where it was determined that multiple logins were being used. Participants could provide basic demographic details but it was not a prerequisite for board participation. The study was approved by The Australian National University Human Research Ethics Committee prior to the launch of *BlueBoard*.

### Sampling

The data was sampled from material collected on *BlueBoard *between 2004 and 2006 from each of the three relevant forums: *Living with Depression*, *Taking Care of Ourselves *(both of which could be browsed by the public) and *Members Only *(restricted to registered members). The three forums comprised a total of 32640 posts, of which the intention was to sample approximately 10%. Therefore, in order to generate a sample of approximately 3000 posts, 335 posts were systematically selected from each year of the three forums. The first post dated 1 March within each year and the subsequent 334 posts were selected. Two of the forums each yielded 1005 posts. However, the third (*Members Only*) forum was developed in 2005; thus, the same sampling strategy yielded 670 posts. The total number of posts analysed was 2680.

### Analysis

The online conversations were examined for evidence of requests for information by participants (reflecting explicit needs) and for reported past or current difficulties (indicative of implicit needs). Although people without personal experience of depression (e.g. carers, friends) could participate in the forum, only the posts of those whose reports indicated they had experienced depression themselves were included in the current analysis. The presence of explicit questions and reported problems were interpreted as indicators of gaps in information.

Thematic analysis of the transcripts was carried out with the assistance of the NVivo 7 qualitative data analysis software package used for coding, annotating, retrieving and reviewing textual data. The data were examined using the data-driven *inductive *approach to thematic analysis, where data are examined for codes and patterns and themes are allowed to emerge from the data [21]. Extracted themes were examined in detail, interpretation of which involved searching the data for concepts, associations and explanations. Instances of explicit questions and the number of people reporting particular types of problems were recorded. One researcher (LB) conducted the analysis and discussed the data with a second researcher (MB) who had moderated the Board and was familiar with the material. The decision not to use multiple raters was based on conclusions that inter-rater reliability of qualitative data does not guarantee reliability or validity of interpretations, and that a methodologically rigorous approach by a single interpreter will result in an accurate reflection of the phenomenon [22].

## Results

### Sample characteristics

A total of 134 board users with a current or a past history of depression contributed to the sample of data analysed. The content of posts suggested that these participants comprised both sexes and a wide age-range and came from both urban and rural populations within and outside Australia.

### Findings

The 2680 posts contained 47 questions (from 46 participants) and 351 reported problems from the total 134 participants. The posts of participants suggested a substantial need for greater information. Some people expressed their information needs explicitly by asking direct questions (n = 47) of other board members. However, in the majority of cases participant needs for information were implicit (n = 351), expressed in terms of experiencing problems and not knowing how to resolve them.

Six broad themes were found. The number of mentions of explicit and implicit need are summarised in Table 1 which is followed by an in-depth description of the nature of these needs. Counts are included where direct questions were asked; where six or more participants reported similar problems; and where quantitative data are useful to illustrate particular points.

**Table 1:** The number of mentions of explicit and implicit needs under thematic areas.

Theme	Explicit needs	Implicit needs
Coping with depression	30	130
Medication	9	35
Professional treatment & services	3	34
Understanding depression	4	26
Disclosure & stigma	1	68
Comorbid mental health problems	0	58

### Themes

#### Coping with depression

A wide range of difficulties associated with coping with depression were observed. Some of these related to problems dealing with issues which were inherent to depression; that is, factors that could be considered indicators or symptoms of depression itself.

Lack of motivation and inhibited enjoyment were reported by many people (17) as being a problem. Some people reported an inability or difficulty with getting out of bed in the morning, whereas the comments of others suggested they were managing everyday demands to some degree but were struggling to find meaning, direction and pleasure in life. Several people reported difficulties finding sufficient motivation to take the necessary steps to help themselves, and explicit questions were evident regarding whether others felt the same way and wanting ideas to resolve such challenges.

"I hear you! I know that if I do my CBT work and start to challenge my negative thoughts that I will start to feel better, but why can I never be bothered to do it??? ... We have a double whammy of an illness, in that to feel better we have to take action, but to take action we have to "feel" like taking action. It's some sort of a cruel joke."

Oversensitivity, negativity and lack of self-esteem and self-loathing were reported by seven people as being problems, including two explicit questions.

"when you have mental illness things that happen around you hit you harder... it hits us harder, it ALL hits us SO MUCH Harder, such small things as not being asked if you'd like a Coffee, so mundane as that can hit you really hard on some of the tough days."

Sleeping difficulties and tiredness were reported by 19 people as being a problem. Problems related to disturbances in sleep onset, maintenance or adequacy. Many people complained of tiredness or exhaustion, some of which - but not all - they attributed to inadequate sleep. The effects of tiredness appeared to cause substantial distress and have a major impact on their lives, and one person asked others explicitly how they dealt with it.

Irritability and not knowing what to aim for in life was the subject of an explicit question, as was how to cope with anxiety and feeling overwhelmed. Several people reported the presence of physical symptoms (i.e. pounding heart, bad headaches, dizzy spells and teeth grinding) that caused them distress and two people asked others for suggestions on how to manage such issues.

Cognitive difficulties and troublesome or obsessive thoughts/feelings were a major problem for many people. A number of people reported struggles with concentration, hypervigilance, racing thoughts and being agitated, and four explicit questions were asked in relation to these. Troublesome thoughts appeared to be particularly upsetting and were reported by 16 people. These were mainly described as being repetitive irrational thoughts which could be difficult to distinguish from reality.

"Things are spiraling beyond my control and I don't like what is happening. My mind keeps processing things and can't stop. Just when I feel that I have distracted it something in the real world screams out a message at me. It might be a song on the radio, someone in my life says something to trigger a thought or whatever. ... I feel like I am losing control and I don't know what to do."

Board rules precluded explicit reporting of suicidal thoughts and self-harm actions. However, there were many subtle references to these problems, with 11 people referring to instances of suicidal thoughts or self-harm. Overwhelming thoughts and despair were reported by another 15 people, five of whom asked explicit questions of others.

"I am struggling so much and it just doesn't make sense... So much to live for but I have lost the will to live. Don't know what to do except to put one foot in front and the next and the next. Please help!"

Difficulties regarding social interactions and loneliness were common and several explicit questions were asked in relation to these issues. The majority of those who reported they felt lonely also recognised their own avoidance of others and withdrawal, but did not know how to resolve the problem. Communication difficulties were also indicated and it appeared that these may underlie or contribute to withdrawal. Some people described a sense of dissociation from others or a need for personal space, with several people referring to being in a 'bubble' including a question asking others if they too felt this way, and reports of feeling 'weird' and 'abnormal'. Interestingly, only one person reported a lack of support from his partner, whereas the majority of people referred to having supportive families.

"Why is it I feel so alone and scared all of the time??? I have a supportive husband and 3 beautiful children but why does that not feel like it is enough ☺ I know I have all of these things as well as more than some people, but why do I feel so alone ??? Is it just me or is it just because I have to deal with this horrible illness that is slowly eating away at me from the inside and is slowly taking a hold of me and I feel it will not let go. I feel so alone."

Nine people referred to experiencing variations in intensity and duration of depression leading to substantial distress, confusion and feelings of hopelessness and a loss of control. The majority of people could not explain the episode and found its unpredictability upsetting, and two explicit questions were asked regarding explanations and advice on "keeping on an even keel".

Many people (23) reported difficulties in relation to work. Some reported difficulties performing their work but continued to function in their jobs to varying degrees. Of these, however, several had received warnings about their performance or felt they were on the borderline of being unable to continue. Even amongst the others there was a lot of uncertainty and evidence of concerns about competency, work-future, and a lack of control. Several people asked explicit questions of others seeking information about eligibility for medical certificates and ideas on how to cope better at work.

"I was wondering how other people out there cope with work on a day to day basis? I have been able to confide in one person at work about my situation, but sometimes it seems impossible to function 'normally' as I don't want the wrong people to find out for fear of discrimination. I feel I can't exactly call in sick due to depression - will a doctor give me a medical certificate? I feel that I am lucky to be able to function at a level where I can hold down a job, but so often it feels like a very precarious position. I would like to hear about other people's experiences and any thoughts you may have."

Some of the 23 had been unable to function in their jobs and had taken leave, resigned, or been encouraged to retire or fired. Of these, some returned to part-time work or found less interesting and satisfying jobs lacking prospects of promotion because it was all they could cope with, and reported the consequence of financial disadvantage.

Other difficulties coping with depression were apparent including agoraphobic tendencies, struggling with the demands of multiple roles, and particularly the overuse of alcohol as 'self-medication'. Also reported was the difficulty dealing with the aftermath of depression: "*... Currently, I do not feel physically depressed, but struggle with the damage that my recent depressive episode has done to my life. Basically, I am trying to rebuild"*, which is non-specific but may refer to issues such as lost job opportunities and relationships.

#### Medication

Amongst the people who had sought professional help the theme of medication appeared to be a critical issue. An area of particular importance was the effectiveness of depression medication. A number of people (7) reported the experience of medication not working adequately, including an explicit question about the effectiveness of a particular type of medication. People reported being unable to find a medication that they felt was effective and expressed concern about the time it can take for people to find a medication that works for them. Even amongst the people who found antidepressants helpful, seven reported that the effect of the medication diminished over time and asked explicit questions of others about this.

"...Recently, I have been feeling worse than ever- is it possible to become "used" to a particular medication, say "toxic" to it, where it just does not seem to be having any impact anymore? ... Could someone offer me an opinion on this please?"

Even when medication was effective in reducing depression symptoms it was not always seen as being beneficial overall, with reported experiences as well as an explicit question indicating some participants felt medication had rendered them unable to experience emotions in a healthy way (i.e. they had ceased to be able to cry or feel joy).

Side-effects of medication were a very common problem, reported by 15 people. The majority had sought medical advice about these but not all were satisfied with the response; others appeared to have been trying to deal with the problems alone. Posts suggested there was a lot of uncertainty about medication and included explicit questions about how long side-effects might last, whether to take medication in the morning or at night, and how to judge whether medication was appropriate or not. Several people reported stopping taking medication because of the extent of side-effects. Such experiences meant that some people had turned to or were considering turning to alternative treatments. This led to direct questioning about alternative options.

"If SAM-e doesn't prove effective as an alternative med, I might try SJW. Did you have any side effects with SJW? I was concerned the dizziness would still occur for me with SJW since I heard it can have similar side effects to prescribed meds. What was your experience with this? It sounds like you're following the path I'd like to be on - lifestyle, diet, alternative therapies, etc. Can you share with me in greater detail what's working for you??"

Discontinuation from medication was also a source of problems for a number of people. Some reported difficulties with prolonged 'withdrawal' symptoms, and presumably in anticipation of such problems, one person explicitly asked others about how to effectively discontinue a particular medication.

#### Professional treatment and services

Reports of difficulties obtaining professional help were observed. Some of these arose from problems negotiating the mental health system such as restricted access to mental health services due to prior incidents with particular professionals and ineligibility to seek assistance from elsewhere.

"the problem is now they won't offer me any help at all. If I turn up to the hospital they are abusive and threatening and refuse to assess me. If I go anywhere else they say they can't see me because I don't live in their area... all I want is some help. ... I'm worried, I'm scared. And I don't know where to go for help. My family and psychiatrist are as stumped as I am. Any clues at all about what I can do? Any suggestions at all would be appreciated"

Lack of knowledge about the mental health system may also hinder access to professional help as was observed in an explicit question regarding self-admission to inpatient care.

Concerns about the unavailability of services were common. In some cases it was due to the lack of services in rural areas where it was necessary to travel substantial distances to see mental health professionals or wait long intervals between visits by professionals. The intervals between appointments were also reported as a problem for some who did not identify as being from a rural population. In other cases, 'unavailability' referred to the inadequacy of existing services:

"Resources for depressed people are often mentioned on related websites and in the blurbs put out by pharm companies. However reality tells a different story. A decent psychologist (psychotherapy) costs a fortune. A psychiatrist costs $150-$200 an hour. Lifeline is staffed by psychology students, some in first year and is not suitable for intelligent, educated, middle aged men. What we are left with is a gp who does not specialise in depression and often has no more knowledge about it then a lay person..."

In addition to restricted availability, the expense of obtaining help from mental health professionals was reported by seven people as being either a hindrance or an insurmountable barrier.

Reservations about the benefits of professional treatment were another common problem - and one which appeared to impede effective management of depression. Several people reported feeling doubtful that professional treatment was helping. Such doubts related to both specific practitioners and a lack of conviction regarding the medical profession or treatment in general.

For some people the focus of the problem was a lack of faith in the capabilities, knowledge, skills and understanding of professionals. The responses of seven people indicated the presence of such concerns, as well as the view that it could be difficult and time-consuming to locate a 'good' professional. There were also indications that attitudes and negative feelings towards professionals could inhibit help-seeking and treatment benefits. Feelings of fear, dislike and a lack of trust in professionals were reported by many people, and there was an explicit question regarding why distrust might exist. Of particular concern were the six reports of negative experiences with professionals which resulted in responses of confusion, anger and a sense of betrayal and subsequent help-seeking avoidance.

#### Understanding depression

Participants' responses indicated they did not have a sufficient understanding of 'depression', particularly in relation to its cause and diagnostic issues. Also evident, however, were other needs related to appropriate help-seeking and recovery. People commonly reported having failed initially to recognise their condition as depression and thus deal with it appropriately. A number of participants reported being depressed for a long time before they were willing to accept they had depression. Others reported they had not realised or acknowledged that they needed help or were reluctant (due to pride or fear) to accept professional help or medication.

Some people expressed a strong desire (including an explicit question) to understand the causes of depression, stating they could not find the answers to this question and that adequate and appropriate resources did not exist.

"Why is our thinking faulty? ... I have to be careful, as I can suddenly slip into depression for no apparent reason and stay locked that way for weeks, Why?... I know I'm not the only one, we as a community of depressed people must all ask questions, Why us. We need answers and help."

Lack of understanding about the complexities of diagnostic issues was also observed.

There appeared to be substantial confusion about diagnostic thinking and terminology, especially regarding the diagnoses of unipolar and bipolar depression. Furthermore, a case of reported confusion about the meaning of test scores from a reputable depression information website suggested that using available resources to clarify diagnoses may be problematic in some cases.

People's posts suggested that the issue of diagnosis causes confusion. In some instances confusion and dissatisfaction had arisen from not being given a clear diagnosis, but there was also discernible confusion or disenchantment with being assigned firm but different diagnoses on different occasions. The desire for accurate diagnoses was reflected in an explicit question about the use of imaging to enable diagnosis, and another explicit question indicated that some people need greater clarification of diagnostic abbreviations and terminology.

The concept of recovery from depression was itself associated with difficulties (and with two explicit questions). Some people raised the issue of recovery as a means of eliciting a sense of hope from others that recovery *is *possible, and/or reported the tendency to self-blame for their failure to achieve it.

"Has anyone here ever recovered from PTSD or depression in general? I mean has anyone ever got to a point in their lives where anxiety, depression no longer lurks in their minds, subconscious or otherwise? I admit I'm asking out of hope... I just wanted to find out if anyone had made it to the other side. If there is a valley of roses at the other end, if there is a treatment that can perform miracles or at least help get me a step up on life, before it passes me by ... "

#### Disclosure and stigma

Problems regarding disclosure and stigma and not knowing how to resolve these issues were mentioned by many participants. Their comments indicated that disclosure about depression and the use of medication for depression was seen to bring with it the risk of negative responses. The posts of 15 people indicated they believed other people (in general, or specific sources including friends and family) did or might respond in a stigmatising manner to their condition. These beliefs appeared to be based primarily on past experiences of negative responses. However, some participants did not refer to specific incidents and some appeared to be reporting *anticipated *concerns.

Fear or experiences of stigma/discrimination in various settings were observed. Of these, fear of stigma in the workplace was the most apparent. Some people referred to a decrease in support over time in the workplace, disrespect or loss of respect by supervisors and co-workers, and the view that disclosure of depression may bring with it the risk of losing career opportunities or result in termination of employment. Overall, it was clear that people were reluctant to disclose their depression and confide in people in the workplace for fear of the consequences, and did not know how to handle disclosure or their work difficulties successfully.

"I'm very worried about the consequences if I tell them about my depression. I believe I was fired from my last job because of it. It's also destroying my confidence because I just feel so stupid all the time and it seems as if no one understands..."

Also reported was discrimination in medical settings that resulted in inadequate or unequal treatment (compared to physical illnesses).

Health professionals themselves were a source of negative responses, with seven people reporting anticipated or experienced negative responses from professionals, leading to fear, confusion and frustration. Some comments indicated that people's previous negative experiences had reduced their future willingness to seek help for their depression.

Self-stigmatising responses also caused substantial distress for a large number of people (seen in the posts of 18 people, including an explicit question). People reported blaming themselves for their condition, seeing it as a personal failing and responding with shame; and one person was angry at her/himself for 'needing' medication. Most often these responses referred solely to internal processes, although one person referred to 'embarrassment' in the presence of other people, and guilt was reported by several people.

"I wish I could stop being so hard on myself, but I blame myself for being this way and no matter what I do or say, that feeling won't stop.... I thought I was stronger than this, but obviously I am not. ... I know that there is a lot of love for me but I don't deserve it... I feel as though I am bringing everyone around me down and that is making me feel worse."

In some cases people recognised that their self criticism was not justified or fair but could not overcome their negative self-responses.

Regardless of the source of stigma it is clear that people felt the need to hide depression, and that this may have become a burden in itself. Seven people referred to 'masking' depression, the demands it made on them and not knowing how to handle them.

"The pain and the sorrow I have to hold in Behind my mask I must hide My feelings I cannot show to the real world... I am hiding again but it's torturing me ... What do I do?"

Except in the above quote, people were not explicit in their need for information about how to deal with stigma. Nonetheless, the extent of reported problems is indicative of a substantial information gap.

#### Comorbid health problems

In addition to depression many people reported the existence of other mental health diagnoses or problems. The predominant comorbid mental health problems were anxiety (28 cases) and substance abuse (9 cases). Other diagnoses included Bipolar Disorder, Post Traumatic Stress Disorder, Schizoaffective Disorder, Borderline Personality Disorder, Obsessive Compulsive Disorder, Dissociative Identity Disorder and eating disorder. Many people reported multiple mental illness diagnoses and their struggles dealing with elements of or the combined burden of their disorders.

"...I have been diagnosed with Major Clinical Depression... also, I have psychosis NOS... Other things have happened to me, which have caused me to develop dissociative identity disorder (DID) and complex post traumatic stress disorder (PTSD). This 'trauma' is not something I can remember and probably not something one discusses...The memories are locked away ... I have been hospitalised a few times."

It is clear that many people with depression also need information about other health issues they might be experiencing.

## Discussion

The instances of explicit questions and the extent of reported problems suggest that people with depression have substantial needs for additional information. Posts indicated numerous problems in relation to coping with depression, medication, services and treatment, recognising and understanding depression, and dealing with disclosure and stigma and comorbid conditions. The relatively modest number of explicitly-expressed needs compared to implicit needs is consistent with findings from other studies of online mutual-help group interactions [20,23] where direct requests for help/information were infrequent compared to disclosure of personal problems in such a way that help is invited.

Directly asking others for information may be relatively infrequent (compared to problem disclosure) but the finding that approximately one-third of participants asked a direct question suggests this approach does not reflect the information-seeking behaviour of only a select few.

Although there were some differences in the information sought through explicit versus implicit approaches (i.e. information needs regarding stigma and comorbid conditions), it was not possible to further differentiate the content areas of the two forms of information-seeking. Consequently, the discussion below considers needs for information as revealed by either or both of these approaches.

### Themes

The most evident of the information needs related to the topic of coping with depression and its impact on people's lives. A number of these needs (e.g. symptom management, how to live with the disorder) correspond with informational needs identified by people with bipolar disorder [7]. The most concerning of these related to the presence of despair and suicidal thoughts. Given the known relationship between depression and suicide [24], the need for information on this topic is not unexpected. However, the current study and the existing literature on suicide suggests that many people do not obtain useful information about coping with suicidal thoughts until after they have come to the attention of crisis teams following an attempt on their lives. Due to the risks involved, these information needs should be addressed as a matter of priority before it is too late.

Other factors which have a major impact on people's lives are lack of motivation and inhibited enjoyment. The number of reported problems and explicit questions indicate that many people need strategies to address these issues. The extent of the need is not surprising considering that these problems are inherent to depression and form part of the diagnostic criteria for the DSM-IV [25]. Consequently, acquiring strategies to increase motivation and boost satisfaction and pleasure may help address both the illness of depression at a core level and the consequences of poor motivation such as poor workplace performance. People experiencing difficulties with motivation (and other difficulties commonly associated with depression) may benefit from being provided with self-help material about behavioural activation strategies or from referral to online cognitive behavioural therapy programs for depression which incorporate such strategies (e.g. the MoodGYM training program; http://www.moodgym.anu.edu.au; e-couch; http://www.e-couch.anu.edu.au).

Sleep difficulties - which may be both a symptom of and/or risk factor for depression [25,26]- were the subject of numerous reports including explicit questions in the current study. Tiredness and inadequate sleep were reported as a major strain for participants and impacted significantly on their well-being. Sleep difficulties may also affect level of functioning and have a major impact on daily activities, with Insomnia having been found to negatively impact on memory, executive function, attention and concentration [27]. It is known that self-help strategies such as limiting stimulants, undertaking physical exercise during the day or early evening, keeping to a regular sleep routine and other cognitive and behavioural strategies (refer to [26]) can be effective in reducing sleep problems and depressive symptoms among people with insomnia [28]. Consequently, these strategies may be helpful for those with a depressive disorder and it is important that people acquire knowledge of them.

Difficulties managing workplace demands featured strongly. Those with depression may benefit substantially from access to information on workplace rights and eligibility for medical leave. Information about strategies for successfully involving supervisors and colleagues to obtain their support, and managing work demands through approaches such as avoiding long hours and over-commitment may also be helpful. Finally, techniques for resolving interpersonal difficulties may be helpful.

Lack of knowledge about depression and its treatment can be a major hindrance to good management. Indications in the current study that people may have difficulty recognising and understanding depression, including its nature, causes, treatment and recovery, reflect the areas of need seen in a study of the questions people with depression ask of their psychiatrist [9]. Further, accepting the need for treatment can be a very slow process. The present findings indicate that lack of understanding about depression inhibits appropriate treatment seeking, and are consistent with previous findings that lack of insight into depression severity and knowledge about mental illness and its treatment contribute substantially to delays in treatment seeking and decision-making [29,30]. Such delays are not inconsequential, ranging between 6 and 8 years for those with mood disorders who do eventually make treatment contact [31]. Clearly, all people in the general community need information about depression and its treatment so that they can seek timely help as appropriate.

People may also require information about where to obtain appropriate treatment, how to access services and appropriate professionals, affordable options, and knowledge about procedures such as self-admission to inpatient services. Those in rural/remote areas may also need advice on how to cope in circumstances of reduced services. This study found that those with depression may avoid professional treatment due to their doubts about the benefits of treatment or lack of confidence in professionals. A study of message board postings on a web-based medical information site found that frustration with physicians was a major theme [12], suggesting that dissatisfaction with mental health professionals may not be uncommon. Consequently, information that addresses typical concerns and increases consumer confidence (e.g. treatment approaches, benefits of treatment, strategies for developing a healthy trusting relationship with professionals) may be helpful in increasing help-seeking and its attendant benefits.

The current findings identified substantial unmet information needs in relation to medication. Such deficits are of particular concern because information about psychiatric medication facilitates adherence [32]. The present analysis suggests that many people who are prescribed antidepressants require a better understanding about the way in which medication operates, medication tolerance and withdrawal, and in particular, knowledge about potential side-effects and strategies for dealing with them. The existence of these gaps in information is consistent with previous conclusions that people may not receive adequate information about medication from healthcare services [11,12]. Such gaps may be due to in part to difficulties in retaining information from the initial consultation [11], people being unaware of their information needs, being aware of needs but not expressing them, or expressing needs but receiving inadequate responses to them. In order to meet their information needs, consumers should be encouraged to ask questions of professionals and discuss the benefits and drawbacks of medications and the possible alternatives, be provided with appropriately targeted take-home material, and have access to other credible sources of information. In relation to this, it is of interest that research involving psychiatric inpatients [33] shows that consumers have preferred delivery modes of medication information with 50%, 17% and 33% preferring oral, written and both oral and written information respectively. This suggests that the provision of information should consider individual preferences.

A range of other information needs was strongly evident implicitly, rather than expressed explicitly. The most striking of these was in relation to the issue of stigma. Reported problems included fear of negative responses from others (including professionals themselves and particularly from people in the workplace) as well as self-stigmatising responses. Overall, and as also seen in other research [34], it was apparent that both forms of stigma may result in inhibited disclosure of depression and reduce the likelihood of receiving appropriate support and treatment. Consequently, consumers may need information that assists them to discover ways of dealing with their reservations and to disclose selectively to reduce the burden of concealing depression. This might include suggestions for dealing with negative or discriminatory responses from others, particularly in relation to the workplace, and information about their rights, anti-discrimination regulations and helpful workplace strategies. Consumers may also benefit from information about strategies for dealing with professionals, including the importance of approaching another professional if the current professional service is unsuitable. Understanding how to manage self-stigma is of particular importance, especially with respect to overcoming self-blame and shame. Nevertheless, the fact remains that stigma is a complex problem and overcoming the stigma associated with mental illness will require comprehensive efforts at the individual, family and community levels, commitment by health professionals and the media, and change to legislation and policy (refer to [35]).

Finally, it appears that many people with depression are dealing with additional mental and physical illnesses. Such conditions may be a cause or consequence of depression or impact further on it. Therefore, in order to deal with depression successfully, consumers also require appropriate information about all illnesses relevant to them, including information about coping with the particular challenges created by multiple conditions.

### Mode of information delivery

In order to satisfy their information needs, particularly those in relation to coping strategies, people with depression need access to a variety of resources. In particular, many people will need more information and support than General Practitioners (GPs) are able to provide due to limitations of GPs' skills and time. However, previous research indicates that patients with depression expect their GP to be the primary source of information and discussion about depression and its treatment and to provide emotional support for decision-making [30]. The availability of other sources and consumer knowledge of their potential contribution may play an important role. It has previously been reported that 32% of a psychiatric inpatient sample preferred input from more than one health care professional on medication information [33]. These findings raise the possibility that people in the community could become amenable to using multiple sources. It may be helpful to raise awareness at a community level of the various potential sources of depression information (e.g. nurses, pharmacists, psychologists, internet) and emphasise the benefits of drawing on a variety of professional sources. In relation to this, health professionals need information to provide to consumers.

The extent to which online support group users with depression disclose their problems and seek information from other board members suggests that these consumers place great importance on the advice of similar others. This tendency was also noted by Powell and Clarke [19], who found that mental health service users particularly value hearing other people's experience of mental health problems. This has implications for the development of resources and delivery of information; for example, information may be most effective when consumers are involved in the development of resources and when presented in the form of similar others' experiences. Furthermore, this approach may help reduce feelings of inadequacy and being different from others as people are reassured to know they are not alone with their difficulties and that their needs are common [19,36]. In addition, reading the stories of those who have experienced the same problems and recovered may be of value to consumers because it instills hope [19]. Once developed, the information should be evaluated by consumers as has been done with patient medication leaflets (refer to [37]).

It is also important to consider why people seek lay opinions online instead of or in addition to seeking information from professionals. Aside from circumstances in which professionals are not accessible or negative interactions are feared (see above), consumers may expect that professionals will not or cannot provide them with what they need. Such beliefs may explain why 21 of the 52 patients in Llewellyn-Jones' [9] study did not ask their psychiatrists any questions. These expectations may occur for a variety of reasons such as perceptions of unequal practitioner-consumer relationships [19], beliefs that the issues fall outside the professional's realm, or a perception that professionals lack the necessary skills or sufficient time. However, even if professionals are willing and able to provide detailed information on a wide variety of topics, consumers place great value on advice from those with first-hand experience who can fully understand and empathise with their problems [19]. They may prefer opinions from lay-consumers that support choice and are empowering rather than information from professionals that is focused on addressing the treatment of depression. Consequently, from the perspective of the consumer, peer-to-peer information-seeking can be very valuable.

There is a substantial amount of information about depression to be found online. Although it might be expected this would be sufficient to meet people's information needs about depression, this is clearly not the case as all participants in the current study had access to internet resources. It is possible that good depression resources exist but are not accessible to lay people; that they exist but are not accessed; that the material is not presented optimally or appropriately, or that the information needed does not exist or is not comprehensive. Further investigation into these possibilities is needed so that appropriate strategies for increasing access to relevant material can be implemented. Inadequacies in online resources about depression are especially concerning because the internet is a significant source of information on mental health for people in the community [18]. Furthermore, high quality, relevant online resources may be critically important in circumstances in which stigma can inhibit information-seeking, where access and cost may restrict professional assistance, and where the disorder has complex and far-reaching consequences that may require long-term management.

### Limitations

The present study interpreted reported problems as indicating areas of implicit unmet need. However, we cannot exclude the possibility that participants possessed sufficient information but - for reasons unknown - failed to use it to resolve their difficulties. It is more likely in fact that the current study *underestimated *the information needs of participants. Firstly, conclusions were based on evidence of explicit or implicit needs, and any existing but unreported problems were not represented. For example, it is possible that there were areas of greater difficulty that people were unwilling to discuss such as sexual difficulties. Secondly, in this study only reports of firsthand personal experiences of problems were counted as instances of problems. This methodology would not have captured problems where the user described a problem without owning or attributing the problem to themselves. It might also be argued that the present findings have limited generalisability because people without access to internet resources may have different information needs. Whilst this may be true, there is no reason to believe they would have a lesser need for information on the themes raised.

Other potential limitations of the current study are that it employed a three-year collection period beginning in 2004 and consumer information needs and internet interaction styles may have changed since that time, it did not report actual demographic data and the analysis was conducted by a single researcher.

## Conclusions

The current study suggests that people with depression have substantial unmet information needs in relation to relation to recognising and understanding depression, services and treatment, medication, coping with depression and its consequences, dealing with disclosure and stigma of depression, and comorbid conditions. Access to online resources and professionals does not ensure that people obtain the information needed, and there is a need to rectify the gaps in provision of information - in both online and offline formats - and the limitations associated with its dissemination. Finally, appropriate information should be targeted not only at those currently experiencing depression, but also at members of the general community who may subsequently develop the disorder.

## Declaration of competing interests

The authors declare that they have no competing interests.

## Abbreviations

*SAM-e*: S-adenosylmethionine; a synthetic form of a compound formed naturally in the body from the essential amino acid methionine and adenosine triphosphate (ATP); used as a supplement. *SJW*: St John's Wort (hypericum perforatum); herbal remedy; *PTSD*: Post Traumatic Stress Disorder.

## Authors' contributions

LB co-designed the study, conducted the analysis and drafted the manuscript. KG established *BlueBoard*, conceived and co-designed the study and critically reviewed and edited the manuscript. MB co-moderated *BlueBoard*, was involved in the acquisition of data and critically reviewed and edited the manuscript content. All authors have read and approved the final manuscript.

## Pre-publication history

The pre-publication history for this paper can be accessed here:

http://www.biomedcentral.com/1471-244X/11/88/prepub
